# Increasing homozygosity is associated with a less open personality

**DOI:** 10.1098/rsbl.2026.0446

**Published:** 2026-11-11

**Authors:** Martin Fieder, Susanne Huber

**Affiliations:** ^1^ Departement of Evolutionary Anthropology, Universität Wien, Vienna, Austria; ^2^ Department of Anthropology, Universität Wien, Vienna, Austria

**Keywords:** personality, homozygosity, inbreeding, group evolution, evolution

## Abstract

This study investigates associations between genome-wide homozygosity and human personality traits. Using data from the Wisconsin Longitudinal Study, comprising approximately 7800 genotyped participants, we calculated runs of homozygosity and inbreeding coefficients from genome-wide SNP data. Personality was assessed with the Midlife Development Inventory. Multivariate and univariate linear models, controlling for sex, birth year and population structure, showed that several genome-wide inbreeding coefficients are significantly associated with some of the Big Five traits. Higher homozygosity was linked to lower openness, lower agreeableness and lower conscientiousness, while extraversion showed a non-significant negative trend and neuroticism showed a non-significant positive trend. These personality patterns may reflect tendencies towards caution and diminished sociability. The findings highlight potential interactions among genetic diversity, social behaviour and personality structure, contributing to the understanding of how evolutionary pressures and population structure shape psychological variation in humans.

## 1. Introduction

Personality psychology identifies several major components of individual variation, including traits, motives, abilities and life narratives. Among these, the Big Five framework—Openness, Conscientiousness, Extraversion, Agreeableness and Neuroticism—has become the dominant model for describing and analysing personality. Recent longitudinal work demonstrates that these traits display moderate-to-high consistency throughout life, while also allowing for systematic developmental change, particularly during early adulthood [1].

Genetic research has shown that personality has a considerable heritable basis. A comprehensive meta-analysis by Vukasović & Bratko [2], encompassing more than 100 000 participants, estimated that around 40% of individual differences in personality can be attributed to genetic factors, with the remainder shaped by environmental influences. Twin studies tend to yield higher heritability estimates than family or adoption studies, indicating methodological effects on genetic estimates.

Genome-wide association research has demonstrated that personality traits are highly polygenic and influenced by numerous genetic variants with individually small effects. Lo *et al.* [3] detected six genomic regions associated with the Big Five dimensions and estimated SNP-based heritability at approximately 0.18, and Lo *et al.* [3] identified several genomic loci associated with the Big Five personality traits. More recent large-scale analyses further expanded this number substantially and highlighted the complex genetic overlap between personality and psychopathology. A meta-analysis by Gupta *et al.* [4], for instance, including up to 682 688 individuals, identified genome-wide significant loci for all five personality dimensions, demonstrating that personality traits are highly polygenic and genetically correlated with psychiatric outcomes including depression, anxiety and risk-taking behaviour. In addition, a meta-analysis by Nagel *et al.* [5], including over 400 000 individuals, identified more than 100 additional loci associated with neuroticism and related affective traits.

Nevertheless, Genome Wide Association Studies (GWAS primarily capture additive effects of individual variants, whereas runs of homozygosity (ROH) analyses may provide complementary insights into the contribution of genome-wide homozygosity, recessive deleterious variants and other non-additive genetic mechanisms underlying personality variation.

Given the strong heritable component of personality, its evolutionary basis has attracted substantial interest. Comparative studies suggest that consistent individual differences—analogous to human personalities—occur across animal taxa [6]. Nettle [7] proposed that each of the Big Five traits reflects adaptive trade-offs that maintain variation within populations: for example, extraversion may enhance mating and exploration success but also increase risk exposure; neuroticism may promote vigilance but at the cost of stress; and openness fosters creativity yet may elevate vulnerability to unusual beliefs or psychopathology. Conscientiousness and agreeableness similarly balance long-term social and fitness benefits against rigidity or reduced self-interest. Frequency-dependent selection [8] further implies that the fitness value of a trait may depend on its prevalence in the population.

Empirical findings support such selective mechanisms. Eaves *et al.* [9] found that human reproductive fitness is linked to interactions between extraversion and neuroticism, indicating that selection may act on personality trait combinations rather than on single personality dimensions. For example, they found that combinations of high extraversion with low neuroticism, or the reverse pattern, were linked to higher reproductive success in women.

Jokela *et al.* [10] similarly reported associations between personality and fertility outcomes in both the Wisconsin Longitudinal Study (WLS) and MIDUS datasets: higher extraversion, openness and lower neuroticism predicted larger family sizes. Studies in non-Western contexts show comparable trends [8]. Accordingly, it could be speculated that directional selection may have led to a substantial increase in these traits in all humans; thus, it does not explain low extraversion, low openness and high neuroticism.

Verweij *et al.* [11] proposed that the maintenance of genetic variation in personality may result from mutation–selection balance—the interplay between the introduction of deleterious mutations and their removal by selection, predicting that mildly deleterious recessive variants are maintained at low frequency in populations. Their results suggest that higher inbreeding is associated with personality characteristics such as lower novelty seeking, reward dependence, and greater harm avoidance. These findings highlight the role of rare variants and non-additive genetic effects in shaping personality diversity. As higher genome-wide homozygosity, reflected in ROHs, increases the likelihood that such variants become homozygous and phenotypically expressed, ROH analyses may reveal subtle effects of inbreeding depression on personality that are not captured by standard GWAS.

Despite growing evidence for genetic influences on personality, research directly linking homozygosity or inbreeding to personality traits remains limited. We therefore examined associations between genome-wide measures of homozygosity and the Big Five personality dimensions in the WLS cohort. Our aim is to test whether higher homozygosity is systematically related to specific personality profiles and to explore the evolutionary implications of such relationships. We perform ROH analyses, as GWAS only identify additive effects of individual variants, whereas ROH analyses capture genome-wide homozygosity resulting from distant parental relatedness. Consequently, ROH approaches provide information about recessive and partially recessive genetic effects that are largely invisible to standard GWAS analyses.

## 2. Methods

The WLS is a long-term study of a random sample of men and women who graduated from Wisconsin high schools in 1957 and their siblings. The WLS panel started out with 10 317 members from the class of 1957. A second sample of 8734 randomly selected siblings of the original graduate panel was recruited for the study. Of these combined samples, 9027 individuals contributed saliva for genetic analysis (4556 individuals are related, as they are members of a family). A total of 713 014 SNPs were genotyped. The WLS genomic data were generated using the Illumina HumanOmniExpress BeadChip, a high-density SNP genotyping array comprising approximately 700 000 genome-wide markers.

Detailed information on individual recruitment, genotyping and quality control can be found at https://researchers.wls.wisc.edu/front-page/data/#genomic. The WLS genetic data are sponsored by the National Institute on Aging (grant nos. R01AG009775, R01AG033285 and R01AG041868) and were conducted by the University of Wisconsin. We have a data use agreement from 30 May 2019 for the project ‘Genome-Wide Association Studies and Runs of Homozygosity’.

## 3. Runs of homozygosity

ROHs are long contiguous stretches of homozygous genotypes that arise when identical chromosomal segments are inherited from a common ancestor (autozygosity). Consequently, ROHs provide a genomic measure of inbreeding and can detect distant shared ancestry even among individuals who are considered unrelated in genealogical records. In general, longer ROHs indicate more recent parental relatedness, whereas shorter ROHs mainly reflect more ancient demographic events, such as historical population bottlenecks or small effective population sizes.

We calculated ROH of the WLS genotypic sample on the basis of the recommendations of Howrigan *et al.* [12] by first removing all SNPs with a minimal allele frequency of lower than 5% (MAF0.05) and performing a ‘moderate SNP pruning’ using a 50 SNP ‘window’, a 5 SNP shift and a VIF of 2. According to Howrigan *et al.* [12], moderate SNP pruning and the threshold of 0.05 MAF have been recommended to improve ROH estimation performance. Although MAF filtering may exclude some homozygous regions, we followed the recommendations of Howrigan *et al.* [12], who demonstrated that moderate pruning and a MAF threshold of 0.05 improve the robustness of ROH estimation and reduce false-positive ROH calls. MAF threshold of 0.05 and pruning resulted in a total of 133 442 SNPs, which we used for the calculations of ROH in a sliding window with a threshold of 50. We performed MAF removal, pruning and ROH calculation in (plink—bfile WLS –indep 50 5 2—out WLS_Pruned; plink—bfile WLS_Pruned_F—homozyg-windowhet0—homozyg-snp 50—out WLS_Pruned_F_ROH).

ROH-calculation produced three measures of ROH each: (i) the total number of homozygote segments (NSEG), (ii) the sum of length of homozygote segments (KB), and (iii) the average length of homozygote segments (KBAVG). The total number of homozygous segments (NSEG) reflects the fragmentation and overall abundance of autozygous regions; the total length of homozygous segments (KB) represents the cumulative burden of genome-wide autozygosity; the average length of homozygous segments (KBAVG) is informative about the temporal origin of inbreeding, with longer average segments indicating more recent common ancestry.

We further calculated FROH, defined as the proportion of the genome contained within ROHs (total ROH length divided by the total autosomal length; as the WLS sample has been sequenced by Illumina array, we used 2770 Mb as the total autosomal length). In addition, we calculated the genome-wide inbreeding coefficient (F_Inbreed), also using Plink: plink –bfile mydata –het –out inbreeding_coeff (F_Inbreed). Furthermore, we used the Principal Component Analysis (PCAs) of the population structure to control for ancestry. PCAs were provided by WLS. We only included individuals of European descent. The construction of the PCA was done by WLS longitudinal and is explained in detail at https://www.ssc.wisc.edu/wlsresearch/documentation/GWAS/Herd_QC_report.pdf.

Unlike GWAS, which identify additive associations between individual common genetic variants and personality traits, ROH analyses examine whether genome-wide autozygosity itself is associated with a phenotype, thereby providing complementary information on recessive deleterious variation, inbreeding depression and non-additive genetic effects that are largely not captured by standard GWAS approaches. However, as ROHs may also arise from linkage disequilibrium rather than recent autozygosity, we repeated the analysis using a minimum ROH length threshold of 1.5 Mb (results are presented in the supplementary material).

The different measures of homozygosity are intercorrelated; all correlations are significant at *p* < 0.0001 (table 1). With a sample size of approximately 7800 individuals, the study had 80% power (*α* = 0.05) to detect correlations as small as *r* = 0.032, indicating that the analyses were well-powered to detect very small genetic effects. Descriptive of ROH measures are shown in table S2 in the supplementary material.

**Table 1.** Correlations among measurements of homozygosity.

	NSEG	KB	KBAVG	Inbred	F_ROH
NSEG	1	0.792	0.602	0.48	0.792
KB		1	0.553	0.583	1
KBAVG			1	0.344	0.553
Inbred				1	0.583
F_ROH					1

## 4. Personality traits

We used the Big Five traits—Extraversion (EXTRA), Agreeableness (AGREE), Conscientiousness (CONST), Neuroticism (NEURO) and Openness (OPEN)—in our analyses because in the WLS, personality was assessed through self-administered questionnaires using the Midlife Development Inventory scales, which measure the Big Five traits. Each trait was measured using 4–6 descriptive adjectives rated on a Likert scale, providing reliable indicators of personality. Participants, drawn from a representative sample of Wisconsin high school graduates from 1957 and their siblings, completed these measures primarily during the 1992–1993, 2003–2005 and 2010–2011 survey waves, when they were in mid- to late adulthood.

In addition, we included the respondent's sex (1 = male, 2 = female) as well as the respondent's year of birth as covariates in the multivariate models calculated. We calculated, in separate models, each of the scaled Big Five variables (NEURO, OPEN, EXTRA, CONST and AGREE) regressing on NSEG, KB, KBAVG, FROH and F_Inbreed, controlling for respondent's sex, scaled year of birth and the scaled first 10 PCA's of the population structure. We excluded relatives closer than first cousins from the analyses. Therefore, in total, we included between 7805 and 7798 individuals (depending on completeness of variables in each model) in our analyses. To further account for genetic relatedness, we included genetic relatedness estimated using KING relationship inference software (https://www.kingrelatedness.com/) as a random effect in the models. Genetic relatedness was coded as 0 = not related and 1 = related as second-degree cousins or closer.

All continuous variables (NSEG, KB, KBAVG, FROH, F_Inbreed, personality traits and year of birth) were z-standardized (mean = 0, s.d. = 1). Fixed-effect estimates are therefore fully standardized regression coefficients (*β*). Models were fitted using lme4 with a Gaussian error distribution. To account for multiple testing, we applied the Benjamini–Hochberg correction to adjust the *p*-values for the 25 tests performed by ordering the *p-*values and comparing each one to an increasingly permissive threshold based on its rank and the total number of tests, rather than applying the same threshold to every test.

## 5. Results

A largely consistent pattern emerged across the measures of ROH and inbreeding (NSEG, KB, KBAVG, F_Inbreed and FROH) (table 2). Openness was the trait most consistently associated with these measures, showing significant negative associations with all predictors after correction for multiple testing. Agreeableness was also significantly negatively associated with all measures except KBAVG after correction for multiple testing, and conscientiousness was significantly negatively associated after correction for multiple testing with NSEG, KB and FROH. These results indicate that individuals with higher levels of ROH and inbreeding tend to score lower on openness, agreeableness and conscientiousness, with the association for openness being particularly robust across all measures. Extraversion also exhibited negative regression coefficients across all predictors, but these associations did not remain significant after correction for multiple testing, whereas regression coefficients for neuroticism were always positive, but never significant.

**Table 2.** Models of the ‘Big five’ regressing on NSEG, KB, KBAVG, F_Inbreed and FROH controlling for sex, birth year and first 10 PCAs for population structure, with kinship included as a random factor (results for the controlling variables are not shown). Beta values, Std. Errors, *p*-values and degree of freedom and Benjamini–Hochberg adjusted *p*-values.

trait	predictor	beta	std. error	*p*	*n*	BH_Adj_P
NEURO	NSEG	0.019	0.012	0.121	7797	0.137
**OPEN**	**NSEG**	**−0.035**	**0.012**	**0.003**	**7798**	**0.016**
EXTRA	NSEG	−0.026	0.012	0.032	7809	0.058
**CONST**	**NSEG**	**−0.032**	**0.012**	**0.007**	**7805**	**0.030**
**AGREE**	**NSEG**	**−0.027**	**0.012**	**0.023**	**7805**	**0.047**
NEURO	KB	0.019	0.012	0.109	7797	0.130
**OPEN**	**KB**	**−0.042**	**0.012**	**0.000**	**7798**	**0.004**
EXTRA	KB	−0.024	0.012	0.042	7809	0.066
**CONST**	**KB**	**−0.027**	**0.012**	**0.020**	**7805**	**0.046**
**AGREE**	**KB**	**−0.028**	**0.011**	**0.015**	**7805**	**0.046**
NEURO	KBAVG	0.005	0.011	0.651	7797	0.703
**OPEN**	**KBAVG**	**−0.026**	**0.011**	**0.020**	**7798**	**0.046**
EXTRA	KBAVG	−0.019	0.011	0.101	7809	0.130
CONST	KBAVG	−0.005	0.011	0.675	7805	0.703
AGREE	KBAVG	0.004	0.011	0.736	7805	0.736
NEURO	Inbred	0.022	0.012	0.074	7797	0.103
**OPEN**	**Inbred**	−**0.040**	**0.012**	**0.001**	**7798**	**0.008**
EXTRA	Inbred	−0.022	0.012	0.067	7809	0.099
CONST	Inbred	−0.026	0.012	0.032	7805	0.058
**AGREE**	**Inbred**	**−0.036**	**0.012**	**0.003**	**7805**	**0.016**
NEURO	FROH	0.019	0.012	0.109	7797	0.130
**OPEN**	**FROH**	**−0.042**	**0.012**	**0.000**	**7798**	**0.004**
EXTRA	FROH	−0.024	0.012	0.042	7809	0.066
**CONST**	**FROH**	**−0.027**	**0.012**	**0.020**	**7805**	**0.046**
**AGREE**	**FROH**	**−0.028**	**0.011**	**0.015**	**7805**	**0.046**

In addition (results not shown), being female was significantly positively associated with neuroticism, extraversion, conscientiousness and agreeableness (and non-significantly positively associated with openness). Being younger was significantly positively associated with neuroticism, openness and extraversion, and significantly negatively associated with agreeableness (and non-significantly negatively associated with conscientiousness).

The results were highly consistent regardless of whether all ROHs or only ROHs longer than 1.5 Mb were included in the analyses (supplementary material, table S1). All genomic inbreeding measures showed the same direction of association with personality traits. In particular, openness, conscientiousness and agreeableness consistently exhibited negative associations with genomic inbreeding, whereas neuroticism showed weak positive associations. The only notable exception was average ROH length (KBAVG), which showed substantially weaker and largely non-significant associations, suggesting that it is less informative than NSEG, KB, FROH or the SNP-based inbreeding coefficient.

## 6. Discussion

The found pattern suggests a link between genetic homozygosity and personality structure. In particular, higher measures of ROH were consistently associated with lower openness and, less consistently, with lower agreeableness and lower conscientiousness. Extraversion exhibited a consistent negative, though (after correction for multiple testing) non-significant association with all measures of homozygosity, whereas neuroticism showed a consistent, but non-significant positive association. Our findings suggest that individuals with higher genomic homozygosity tend to exhibit personality profiles characterized by lower openness to novelty, diminished sociability and reduced cooperative tendencies. By contrast, the slight positive but non-significant association with neuroticism could reflect a tendency of heightened emotional sensitivity or stress reactivity. Together, these patterns may point towards behavioural dispositions emphasizing caution, familiarity and social closure rather than exploration or engagement with out-groups.

As characteristics such as openness, agreeableness and conscientiousness are commonly viewed as more desirable, one might expect selection to favour higher levels of these traits, leaving their persistence at lower levels unexplained. One possible evolutionary interpretation for their persistence at lower levels is that while those characteristics are often viewed as less adaptive in modern, diverse societies, they may have been advantageous in ancestral contexts marked by ecological threats or intense intergroup conflict. Accordingly, such variation in these traits may reflect evolutionary trade-offs and fluctuating selection across changing ecological and social environments [7,13] or different fitness consequences across ecological niches [14]. Supporting this view, Gurven *et al.* [15] found that associations between personality and reproductive fitness in a small-scale subsistence population varied by sex and socioecological context. Moreover, historically high pathogen prevalence has been associated with lower openness and extraversion [16], while recurrent intergroup conflict may have favoured more parochial, in-group-oriented social strategies [17]. Thus, under particular ancestral conditions, more cautious or less socially exploratory phenotypes may have conferred advantages.

Hence, increased homozygosity may have involved a trade-off: the greater expression of deleterious alleles may have imposed fitness costs, but associated behavioural tendencies such as lower openness may have conferred context-dependent advantages under conditions of hostile intergroup interactions, high parasite load, and elevated infectious risk. Consistent with this view, intergroup behaviour such as tolerance or avoidance is highly flexible and responsive to socioecological costs and benefits [13,18], and recurrent intergroup conflict may have favoured group-beneficial and parochial social behaviour [17]. In addition, according to Schaller & Murray [16] and Kramer & Bressan [19], infection threat can promote social withdrawal, lower openness, stronger in-group cohesion and avoidance of unfamiliar individuals.

Conversely, more open individuals may have benefited in stable or resource-rich environments where social exploration was less risky. This interpretation resonates with earlier findings linking homozygosity to harm avoidance [11]. In such environments, personality traits promoting out-group interaction—such as openness or extraversion—could facilitate gene flow and enhance genetic diversity, albeit with higher exposure to external risks. Sex differences may further modulate these dynamics. Women have often been the primary agents of intergroup gene flow through exogamous marriage [20], potentially explaining stronger xenophilic tendencies observed across cultures. Men, more frequently involved in intergroup competition [21], may have experienced greater selection for parochial behavioural profiles. Thus, our interpretation does not imply that one personality profile was universally advantageous. Instead, fluctuating ecological and social environments, together with the complex genetic architecture of polygenic traits, may have maintained personality variation rather than driving directional selection towards a single phenotype. Generally, the evolution of complex polygenic traits such as personality likely involved multiple evolutionary processes beyond those associated with inbreeding and homozygosity, and the context-dependent advantages proposed here represent only one possible component of this broader evolutionary process. For instance, variation in polygenic traits may be maintained by mutation–selection balance and stabilizing selection [22], while their response to selection can be constrained by complex genetic architecture and pleiotropy [23] and shaped by heterogeneous environments favouring different local phenotypic optima [13].

In line with gene–culture coevolution [24], our results are also consistent with evidence that in-group preferences and restricted mating can contribute to genetic differentiation. Previous research reported that stronger ethnic in-group preferences have been associated with higher genomic homozygosity [25], potentially because assortative mating within culturally similar groups modestly increases homozygosity over generations.

Alternatively, the observed relationships could also reflect the mutation–selection balance model. If environmental threats encourage population isolation, higher homozygosity may accumulate, leading to greater expression of less advantageous alleles and associated personality profiles. Thus, these personality profiles could reflect the phenotypic consequences of increased homozygosity rather than evidence that such traits were directly favoured by natural selection. However, as the effects are rather small, we also have to take into account the possibility that the relationship between homozygosity and personality may simply be a correlation without an underlying causation like selection.

Unlike GWAS, ROH analyses can reveal the cumulative impact of recessive alleles that become homozygous through shared ancestry; the found associations further suggest that genome-wide homozygosity may modestly contribute to personality variation through non-additive genetic mechanisms, such as the expression of recessive alleles. Stronger associations for NSEG and KB than for KBAVG may indicate that the cumulative burden of homozygosity rather than very recent inbreeding is more relevant. However, given the small effect sizes, these findings should be interpreted cautiously and require replication.

In summary, our findings indicate that genome-wide homozygosity is linked to variation in major personality dimensions, particularly those associated with social openness and cooperation. The observed associations are consistent with the hypothesis that traits associated with lower openness, lower agreeableness and lower conscientiousness may be influenced by partially recessive genetic variants revealed through increased homozygosity. Such patterns are compatible with several complementary evolutionary explanations such as mutation–selection balance and inbreeding depression models, although evolutionary explanations involving social ecology cannot be excluded. Future research combining genetic, behavioural and cross-cultural data will be valuable for testing how these mechanisms jointly shape the evolution of human personality.

## 7. Limitations

The observed associations were small and explain only a minor proportion of personality variation. Furthermore, ROH measures provide an indirect estimate of genome-wide homozygosity and do not identify specific causal variants or biological pathways. Finally, the WLS sample is restricted to individuals of predominantly European ancestry from a single birth cohort, which may limit the generalizability of the findings.

## Supplementary Material



## Data Availability

Data are available as supplementary material; all data are available free of charge from the WLS upon request at https://researchers.wls.wisc.edu/front-page/data/#genomic and after approval by the local Institutional Review Board (IRB). Supplementary material is available online [26].
