# Supplementary material for: Increasing homozygosity is associated with a less open personality
Source: Biol Lett. 2026 Nov 11;22(11):20260446. doi: 10.1098/rsbl.2026.0446 (PMC13752741; doi:10.1098/rsbl.2026.0446)
Supplement: Supplementary file 1 [file rsbl.2026.0446.zip › rsbl.2026.0446/rsbl20260446_si_002.docx]

**Supplement**

**Table S1)** Models of the “Big Five” on NSEG, KB, KBAVG,F_Inbreed, F_ROH controlling for sex and birth year and first 10 PCA’s for population structure and with kinship included as random factor, only for individuals with longer strechtes of autozygoisty that 1.5MB. Beta vlues, Std. Errors, P- Values.

| **Trait** | **Predictor** | **Estimate** | **Std. Error** | **P value** |
| --- | --- | --- | --- | --- |
| NEURO | NSEG | 0.03597 | 0.01474 | 0.0147 |
| OPEN | NSEG | -0.04063 | 0.01458 | 0.005334 |
| EXTRA | NSEG | -0.02176 | 0.01453 | 0.134516 |
| CONST | NSEG | -0.04989 | 0.01453 | 0.000601 |
| AGREE | NSEG | -0.04026 | 0.0144 | 0.00521 |
| NEURO | KB | 0.02314 | 0.01232 | 0.0603 |
| OPEN | KB | -0.04261 | 0.01206 | 0.000414 |
| EXTRA | KB | -0.02092 | 0.01203 | 0.082003 |
| CONST | KB | -0.03125 | 0.01203 | 0.00941 |
| AGREE | KB | -0.02946 | 0.01192 | 0.0135 |
| NEURO | KBAVG | 0.02161 | 0.0162 | 0.1825 |
| OPEN | KBAVG | -0.03346 | 0.01618 | 0.038676 |
| EXTRA | KBAVG | -0.01317 | 0.01612 | 0.414202 |
| CONST | KBAVG | -0.00808 | 0.01614 | 0.6165 |
| AGREE | KBAVG | 0.0075 | 0.01599 | 0.63927 |
| NEURO | Inbred | 0.02767 | 0.01456 | 0.0574 |
| OPEN | Inbred | -0.05182 | 0.01436 | 0.000312 |
| EXTRA | Inbred | -0.01894 | 0.01434 | 0.186631 |
| CONST | Inbred | -0.03302 | 0.01434 | 0.0213 |
| AGREE | Inbred | -0.04404 | 0.01421 | 0.00195 |
| NEURO | F_ROH | 0.02896 | 0.01541 | 0.0603 |
| OPEN | F_ROH | -0.05332 | 0.01509 | 0.000414 |
| EXTRA | F_ROH | -0.02619 | 0.01505 | 0.082003 |
| CONST | F_ROH | -0.03911 | 0.01506 | 0.00941 |
| AGREE | F_ROH | -0.03687 | 0.01492 | 0.0135 |

Table S2) Descriptive Statistics ROH measures

|  | Mean | SD | Median | P5.5% | P25.25% | P75.75% | P95.95% | Min | Max |
| --- | --- | --- | --- | --- | --- | --- | --- | --- | --- |
| NSEG | 1.576 | 1.868 | 1 | 0 | 0 | 2 | 4 | 0 | 47 |
| KB | 3772.21 | 12995.89 | 2184.715 | 0 | 0 | 4119.992 | 9189.88 | 0 | 536690 |
| KBAVG | 1482.89 | 1276.891 | 1520.745 | 0 | 0 | 2007.632 | 3240.02 | 0 | 15822.5 |
| Inbred | 0.002 | 0.008 | 0.002 | -0.008 | -0.002 | 0.006 | 0.011 | -0.069 | 0.2 |
